# The gut microbiota as a booster for radiotherapy: novel insights into radio-protection and radiation injury

**DOI:** 10.1186/s40164-023-00410-5

**Published:** 2023-05-22

**Authors:** Yuxi Yi, Weiqing Lu, Lijun Shen, Yang Wu, Zhen Zhang

**Affiliations:** 1 Department of Radiation Oncology, Fudan University Shanghai Cancer Center, Shanghai, China; 2 Department of Oncology, Shanghai Medical College, Fudan University, Shanghai, China; 3 Key Laboratory of Medical Molecular Virology (MOE/NHC/CAMS), Shanghai Frontiers Science Center of Pathogenic Microorganisms and Infection, School of Basic Medical Sciences, Shanghai Medical College, Fudan University, Shanghai, China; 4 Shanghai Clinical Research Center for Radiation Oncology, Shanghai, China; 5 Shanghai Key Laboratory of Radiation Oncology, Shanghai, China

**Keywords:** Gut microbiota, Radio-protection, *Lactobacillus*, *Akkermansia*, Short chain fatty acid, Microbiota transplantation, Probiotic, Toll-like receptor

## Abstract

Approximately 60–80% of cancer patients treated with abdominopelvic radiotherapy suffer post-radiotherapy toxicities including radiation enteropathy and myelosuppression. Effective preventive and therapeutic strategies are lacking for such radiation injury. The gut microbiota holds high investigational value for deepening our understanding of the pathogenesis of radiation injury, especially radiation enteropathy which resembles inflammatory bowel disease pathophysiology and for facilitating personalized medicine by providing safer therapies tailored for cancer patients. Preclinical and clinical data consistently support that gut microbiota components including lactate-producers, SCFA-producers, indole compound-producers and *Akkermansia* impose intestinal and hematopoietic radio-protection. These features serve as potential predictive biomarkers for radiation injury, together with the microbial diversity which robustly predicts milder post-radiotherapy toxicities in multiple types of cancer. The accordingly developed manipulation strategies including selective microbiota transplantation, probiotics, purified functional metabolites and ligands to microbe-host interactive pathways are promising radio-protectors and radio-mitigators that merit extensive validation in clinical trials. With massive mechanistic investigations and pilot clinical trials reinforcing its translational value the gut microbiota may boost the prediction, prevention and mitigation of radiation injury. In this review, we summarize the state-of-the-art landmark researches related with radio-protection to provide illuminating insights for oncologists, gastroenterologists and laboratory scientists interested in this overlooked complexed disorder.

## Background

As the largest symbiotic reservoir in the human body, the gut harbors a plethora of microorganisms and functional genes, molecules and metabolites, which make up the gut microbiota of high research value in the pathophysiology of the host, especially in aspects of inflammation, metabolism and immunomodulation. Recently, groundbreaking studies on the gut microbiota influencing the anticancer immune response have generated widespread interest in this “secondary organ” in the field of oncology. The addition of “polymorphic microbiomes” to the newest version of cancer hallmarks [1] also highlights its importance in tumorigenesis.

Radiotherapy is a compulsory modality for over 50% of all cancer patients with a curative role in 25% of cancers [2], by precisely delivering high-energy ionizing radiation to eradicate malignant cells. However, enterocytes and hematopoietic cells are also highly sensitive to radiation because of their rapid renewal rates. Despite advanced delivery techniques to protect normal tissues, toxicities including radiation enteropathy (RE) and myelosuppression are common in patients treated with radiotherapy for abdominopelvic tumors. It is estimated that over half of all cancer survivors are patients with abdominopelvic tumors, and approximately 60–80% of those receiving abdominopelvic radiotherapy develop varying degrees of acute RE within three months from the first radiation, while 5–20% of them later develop chronic RE [2, 3]. This estimation is conservative due to unsatisfactory follow-up, and the prevalence of post-radiotherapy gastrointestinal dysfunction is believed to exceed that of inflammatory bowel disease (IBD) in US [2]. Although acute RE is self-limited, severe symptoms such as dehydration, electrolyte imbalance and enteric infection occurring in neutropenic cases can lead to treatment suspension and even death. Delayed symptoms like chronic blood loss and severe stricture or perforation, are irreversible and life-threatening occasionally, which constitute major dose-limiting factors for radiotherapy. Concurrent cytotoxic chemotherapy and biotherapy further exacerbate the toxicities, impede completion of therapy and compromise tumor control. Therefore, radio-protection deserves high attention to ensure safety, efficacy and adherence of radiotherapy.

However, no reliable predictive tools or preventive strategies have been established currently for radiation injury. Symptomatic treatment remains the mainstream salvage option. Amifostine is the only medication approved by the U.S. Food and Drug Administration (FDA) for preventing radiation-induced toxicity, whereas severe collateral side effects and narrow efficacy time window limit its widespread use [2]. To our knowledge, the gut microbiota is an area with intensive research to address the unmet needs of radio-protection. Reviews focusing on the interplay between gut microbiota and gastrointestinal mucositis induced by radiotherapy or chemotherapy have been published yet neither covering microbial influences on hematopoietic impairment nor a summary of mechanistic insights. To fill in the knowledge gap and to promote deeper understanding on the radiation injury pathogenesis for designing novel predictive, prophylactic and therapeutic strategies, we reviewed updated studies in this field especially mechanism-driven laboratory findings. For the convenience of the readership, the definitions of important terms in this review are summarized in Table 1.

**Table 1:** Definitions of key terms

Key term	Definition
ARE	Gastrointestinal symptoms that occur within 12 weeks of abdominopelvic radiotherapy in cancer patients, including nausea, vomiting, diarrhea and abdominal pain, etc. Featured by progressive cell impairment and inflammation, the rapidly renewing intestinal epithelium is the major target and thus symptoms are reversible and short-lasting
CRE	Gastrointestinal symptoms that occur after 12 weeks until years after abdominopelvic radiotherapy in cancer patients, including dysmotility, malabsorption, strictures, perforation and bleeding, etc. Featured by mucosal atrophy, vascular sclerosis and progressive fibrosis, the slowly renewing intestinal parenchymal wall is involved, and thus symptoms are usually irreversible
Gut microbiota	Microorganisms habituating in the gastrointestinal tract of host, including bacteria, archaea, fungi and viruses, with broad impacts on metabolism and pathophysiology of host
MT	The transfer of microbial content from a healthy donor into the gastrointestinal tract of a diseased recipient, and can be classified into faecal MT, oral MT, selective MT according to the sources
Probiotic	Live microorganisms that, when consumed in adequate amounts, beneficially affect the host by direct bacterial-host interactions or derived metabolites
Prebiotic	Nondigestible ingredients e.g., fibers, that reach the colon and promote growth of specific colonic bacteria to beneficially improve host health
Synbiotic	Combination of probiotics and prebiotics and are believed to be more efficient in conferring health benefits

*ARE* Acute radiation enteropathy, *CRE* Chronic radiation enteropathy, *MT* Microbiota transplantation

## Preclinical evidence links the gut microbiota to radiation injury

### Causal linkage established from deprivation, supplementation and interventional assays

Elegantly-designed experimental studies establish the strong linkage between the gut microbiota and radiation injury. Resistance to lethal dose total body radiation (TBI) in germ-free mice compared to conventionally-raised mice has been reported since 1950s [4]. In line with this finding, pre-treatment with antibiotics to clear the gut microbiota also significantly improves survival in mice exposed to sublethal dose TBI [5] and accelerates recovery of oral mucositis after snout radiation [6]. Moreover, Fan et al.conducted a series of works by introducing a third factor which interacts with the gut microbiota and thus affects radio-sensitivity of normal tissues to reveal the bridging role of gut microbiota in radiation injury. In their reports, circadian rhythm disturbances [7] and food additive polysorbate-80 [8] disturbing the gut microbiota lead to radiation injury deterioration. Whereas modified diets [9, 10], anti-cholesterol agent simvastatin [9] and anti-oxidant molecules [11–14] mitigate radiation injury in a microbiota-dependent manner. Noteworthy, a series of gender-specific phenotypes in relation with gut microbiota interplaying with radiation injury is repeatedly reported in Fan’s animal models [5, 9, 10]whose underlying mechanism is unclear. Whether similar manifestations exist in human as a possible explanation for the sex differences in treatment efficacy and toxicity [15] remain to be investigated. Nevertheless, such a phenomenon reminds us that gender is of concern in unravelling the role of gut microbiota in radiation injury. We summarize the animal models used in experimental studies (Table 2**)**, noticing that most studies chose male CB57BL/J6 (B6) mice in this field.

**Table 2:** Preclinical studies establishing the cause-and-effect role of gut microbiota in radiation injury

References	Animal model	Key techniques	Major endpoints	Conclusions and highlights
Onoue, Japan, [16]	ICR mice, M, n = 10–42/group	TBI: 20 Gy Oral administration of single strain	Survival; histopathology of multiple organs	The first study distinguishing beneficial microorganisms from harmful ones based on the observation of radio-resistance in germ-free mice
Crawford And Gordon, US, [4]	FVB/N, B6 mice, M/F, n = 3–27/group	TBI: 10–22 Gy Bacteria culture	Survival; histopathology of intestine	The radio-protective fasting-induced adipose factor is suppressed by the gut microbiota although the specific microbe(s) responsible were not identified
Lam, US, [19]	Wistar rats, M, n = 5/group	TBI: 10, 18 Gy/6Fx Feaces for 16S rRNA sequencing	NA	The first study establishing microbiota-based acute and chronic ratios as effective biomarkers of prior radiation exposure
Fan, China, [7]	B6 mice, M, n = 3–10/group	TAI: 5 Gy Feaces for 16S rRNA sequencing	Survival	Circadian rhythm disorder interacts with gut microbiota to augment radiation injury
Fan, China, [5]	B6 mice, M/F, n = 4–18/group	TBI: 6.5 Gy Feaces for 16S rRNA sequencing + intestine for RNA sequencing	Survival; histopathology of intestine + circulating blood cell counting + spleen weight	Faecal microbiota transplantation is radio-protective without accelerating tumor growth
Fan, China, [13]	B6 mice, M, n = 4–20/group	TAI: 15 Gy Feaces for 16S rRNA sequencing + intestine for microRNA assay	Survival; histopathology of intestine + circulating blood cell counting	Radio-protective hydrogen-water restores radiation-induced gut dysbiosis
Gerassy, Israel, [17]	B6 mice, F, n = 8–33/group Germ-free Swiss Webster mice† for faecal MT assay	Rectum brachytherapy: 22 Gy/4Fx Feaces and colonic tissues for 16S rRNA sequencing + cytokine analysis	Survival; histopathology of intestine	The pro-inflammatory dysbiosis induced by radiation is transmissible via faecal microbiota transplantation and renders radio-sensitivity to intestine
Tian, China, [22]	SD rats^a^, n = 30/group	TBI: 0,4,8,12 Gy Feaces for 16S rRNA sequencing	Survival; histopathology of intestine	The first study showing the dose-dependent microbiota features in association with radiation injury in rodent models
Carbonero, US, [21]	Gottingen minipig, M, n = 7–13/group Chinese rhesus macaques, M, n = 12–16/group	TBI: 1.65–2.25 Gy for minipig; 5.9–7.7 Gy for macaque Feaces for 16S rRNA sequencing	Survival	The first study showing the dose-dependent microbiota features in association with post-radiation survival in large primate models
Kweon, Korea, [55]	B6, ICR mice, M/F, n = 3–5/group	TBI: 10 Gy or 12 Gy Gavage with lactate-producing microbes or conditioned medium or lactate + intestine for lactate measurement	Survival; histopathology of intestine + organoid measurement	Probiotics-derived lactate imposes intestinal radio-protection by activating Wnt/β-catenin in intestinal stem cells to accelerate epithelial repairment
MA Ciorba, US, [53], [54]	B6^b^, BALB/c^a^, n = 4–10/group	TBI: 12 Gy, TAI: 28-32 Gy/7-8Fx Gavage with live or heat-killed *Lactobacillus* or *Lactobacillus*-conditioned medium Luminal microbial analysis using qPCR	Survival; histopathology of intestine + circulating lymphocyte & hematopoietic stem cell counting	*Lactobacillus*-derived lipoteichoic acid imposes intestinal radio-protection without compromising anticancer efficacy by activating EGF pathway in intestinal stem cells
Fan, China, [11]	B6 mice, M, n = 3–10/group	TAI: 15 Gy Feaces for 16S rRNA sequencing	Survival; histopathology of intestine + circulating blood cell & bone marrow cell counting	Radio-protective 3,3’-diindolylmethane restores radiation-induced gut dysbiosis
Li, China, [12]	B6 mice, M, n = 3–15/group	TBI: 9 Gy Feaces for 16S rRNA sequencing + intestine for RNA sequencing	Survival; histopathology of intestine + organoid culture	Radio-protective VND3207 restores radiation-induced gut dysbiosis
Fan, China, [9]	B6 mice M/F, n = 5–24/group	TBI: 7 Gy for survival; 4 Gy for hematopoietic toxicity; TAI: 12 Gy for gastrointestinal toxicity; Feaces for 16S rRNA sequencing + intestine for RNA sequencing	Survival; histopathology of intestine + spleen & thymus weight and blood inflammatory markers	Simvastatin and high-fat diet is radio-protective in male and female mice respectively, which relies on the existence of the sex-specific gut microbiota
Fan, China, [8]	B6 mice M, n = 12/group	TAI: 12-15 Gy Feaces for 16S rRNA sequencing & SCFA quantification (LC)	Survival; histopathology of intestine	Polysorbate-80 aggravates acute RE by altering the gut microbiota and decreasing butyrate level; post-radiation administration of butyrate reverses the effects of polysorbate-80
Fan, China, [42]	B6 mice, M/F, n = 3–12/group	TBI: 4-7 Gy; TAI: 12-15 Gy Feaces for 16S rRNA sequencing & SCFA quantification (LC) + intestine for peptide quantification (MS)	Survival; histopathology of intestine + spleen & thymus weight and blood inflammatory markers	Microbiota-derived valerate alleviates radiation injury by up-regulating AML1/KRT1 which is down-regulated after radiation and is radio-protective
Fan, China, [43]	B6 mice & Balb/c nude mice, M/F, n = 6–30/group	TBI: 7.2 Gy for survival; 4 Gy for hematopoietic toxicity; TAI: 12 Gy for gastrointestinal toxicity; Feaces for 16S rRNA sequencing & untargeted metabolomics (LC/MS) + intestine for peptide quantification (MS)	Survival; histopathology of intestine + spleen & thymus weight and blood inflammatory markers	Microbiota-derived IPA alleviates radiation injury by re-activating the PXR-ACBP pathway without compromising anticancer efficacy
Guo, US, [47]	B6 mice, M/F, n = 4–33/group	TBI: 8.0–9.2 Gy Feaces for 16S rRNA sequencing & SCFA quantification (GC) & untargeted metabolomics (LC)	Survival; histopathology of intestines, spleen & bone marrow	Microbiota-derived SCFAs and tryptophan metabolites confer hematopoietic and gastrointestinal radio-protection without compromising anticancer efficacy
Tian, China, [20]	B6 mice, M, n = 5–10/group	TBI: 0,4,8,12 Gy for dose-dependent assay and 9 Gy for probiotic assay; Feaces for 16S rRNA sequencing	Survival; histopathology of intestine	A longitudinal study demonstrating that microbial quantifications at day3.5 after radiotherapy provide biomarkers for radio-dosimetry and radiation injury
Fan, China, [14]	B6 mice, M, n = 5–8/group	Total chest irradiation: 15 Gy Feaces for 16S rRNA sequencing + LC/MS	Body weight, histopathology of lung and heart	Radio-protection of L-Histidine relies on the existence of the gut microbiota
Fan, China, [10]	B6 mice M/F, n = /group	TBI: 5 Gy; TAI: 12 Gy Feaces for 16S rRNA sequencing	Survival; histopathology of intestine + circulating blood cell counting	Radio-protection of caloric-restriction diet relies on the existence of the gut microbiota
Kweon, Korea, [63]	B6, F, n = 3–5/group	TBI: 10 Gy Gavage with *Akkermansia* or conditioned medium + Cecal contents for SCFA quantification (LC/MS)	Survival; histopathology of intestine + organoid measurement	*Akkermansia*-derived SCFAs impose intestinal radio-protection by activating Wnt/β-catenin in intestinal stem cells to promote proliferation and differentiation
Epperly, US, [23]	B6, BALB/c, sv129 mice, M, n = 10–20/group	TBI: 9.25 Gy Feaces for 16S rRNA sequencing and qPCR validation	Survival	*Akkermansia muciniphila* improves post-radiation survival in TBI mice
Epperly, US, [24]	B6 mice, F, n = 12/group	TBI: 9.25 Gy; TAI: 19.75 Gy; Feaces for 16S rRNA sequencing	Survival; inflammatory protein expression + bone marrow cells counting	Engineered IL-22- producing microbe ameliorates radiation injury
Epperly, US, [25]	B6 mice, M, n = 10–15/group	TBI: 9.25 Gy Feaces for 16S rRNA sequencing	Survival	Inclusion of microbiota information improves the predictability of survival when controlling for administration of radiation mitigators
Dar, US, [52]	B6 mice, F, n = 3–5/group	TBI: 9.25 Gy Feaces for bacterium counting + intestine for lipidomics (LC/MS)	Survival; histopathology of intestine	*Pseudomonas aeruginosa*-derived 15-lipoxygenase increases lipid peroxidation and ferroptosis, thereby exacerbating local inflammation and radiation injury

*M* Male, *F* Female, *TBI* Total body irradiation, *TAI* Total abdominal irradiation, *NA* Not applicable, *Fx* Fractions, *Qpcr* Quantitative polymerase chain reaction, *EGF* Epidermal growth factor, *SCFA* Short chain fatty acid, *LC* Liquid chromatography, *MS* Mass spectrometry, *GC* Gas chromatography, *KRT1* Keratin, type II cytoskeletal 1, *IPA* Indole 3-propionic acid, *PXR* Pregnane X receptor, *ACBP* Acyl-CoA-binding protein

^a^Gender not mentioned

^b^Female mice were preferentially used

In addition to associations from deprivation and interventional assays, selective microbiota supplementation assays provide more robust evidence. For example, mono-association experiments revealed that *Fusobacterium* sp., *Streptococcus faecalis*, *Escherichia coli* and *Pseudomonas* sp. respectively contributes to radiation injury augmentation, whereas *Clostridium* sp., *Bifidobacterium pseudolongum* and *Lactobacillus acidophilus* contributes to injury amelioration [16]. Furthermore, repeated transplantations of feaces from gender-matched healthy mice after radiation efficiently mitigate intestinal and hematopoietic toxicity by enhancing angiogenesis and maintaining gut barrier integrity of the radiated mice [5]. On the other hand, transplantation of dysbiosis feaces from radiated mice [17] or from polysorbate-80-treated mice [8] before radiation transmits susceptibility to radiation injury to the recipients.

### Reinforced predictive and prognostic value by microbial dynamics during radiation

Compared to cross-sectional studies, repeated sampling in a longitudinal design has higher statistical power, and some studies associate the microbial dynamics throughout radiotherapy with radio-dosimetry [18–22] and radiation injury [17, 23–25]. Reportedly, abdominal dose of  > 1.5 Gy is a risk factor of RE and the dose threshold for gastrointestinal lining loss is 6 Gy [26]. The threshold for hematopoietic dysfunction is 0.5 Gy and the exquisitely radio-sensitive lymphocytes are maximally depressed when exposed to radiation of ~ 2 Gy, which is the standard fractionated regimen adopted in the clinic [27]. According to historic reports and our clinical experience, patients typically present gastrointestinal symptoms since the first or second week of radiotherapy [26] and may develop progressive myelosuppression almost immediately within the first week of radiotherapy [27]. Therefore, timely identification of high-risk population (> 2 Gy absorbed dose) in need of medical intervention is critical both for ensuring safety of radiotherapy in clinical settings and for appropriate allocation of medical resources after accidental nuclear exposure. To this end, Lam et al. [19] developed two indices respectively named as “acute ratio”, which climbs within the first week after radiation, and “chronic ratio” which increases from the second till the third week, based on murine faecal microbes whose abundances are significantly and persistently altered after TBI. The two indices effectively determine exposure to single-fraction of 10 Gy radiation and multiple-fractions of 18 Gy/6Fx radiation. Interested in the feasibility of microbiota as radio-dosimetry, Tian’s group [20] using rodent models reported linear correlations between radiation dose and microbial abundances measured at day 3.5 following radiation, including negative association with *Eubacterium xylanophilum* and positive association with *Escherichia Shigella* (*p*-value = 0.046 and 0.026, respectively). These microbial abundances also parallel with the post-radiation lethality and intestinal histopathologic features. Likewise, Carbonero et al. [21] found in minipig and macaque models significant correlations between radiation dose and microbial abundances measured at day 3 following TBI, including the positive association with butyrate-producing *Clostridium* XIVa as well as the negative association with *Prevotella*, and the abundance of *Prevotella* is suggested as a potential positive prognostic biomarker of post-radiation survival.

A further step made by Epperly et al. [25] is the inclusion of microbiota information into post-radiation survival models which efficiently improves the predictability. They set the repeatedly measured microbiota abundances as time-varying covariates combined with static covariates of several radiation mitigators including granulocyte-colony stimulating factor (G-CSF), programmed cell death inhibitors JP4-039, necrostatin-1 and baicalein in a Cox proportional hazards model. Results showed that both the abundance of *Lactobacillus* (HR = 0.15, *p*-value = 0.001) and butyrate-producing *Ruminococcus* (HR = 0.85, *p*-value = 0.04) are independently robust predictors of increased survival, whose addition into the model improves the concordance index, even outweighing the significance of therapeutic G-CSF or the triple regimen of JP4-039/baicalein/necrostatin-1 (*p*-value = 0.08 and 0.09, respectively). Surprisingly, abundance of *Akkermansia* is a deleterious predictor of survival (HR = 2, *p*-value = 0.046) in experiments incorporating G-CSF although the prognostic value of *Akkermansia* is insignificant in experiments using programmed cell death inhibitors only. Given the marginally significantly decrease in *Akkermansia* in survivors treated with G-CSF, the possibility that the interplay between *Akkermansia* and G-CSF renders this microbe the unexpected correlation with radiation-induced death needs to be clarified.

To summarize, preclinical results strongly support that the gut microbiota is closely involved into the development of radiation injury. Of note, over 96% of the microbial biomarkers identified in murine faeces have been matched to genera existing in human [19], highlighting the translational value of these laboratory results.

## Bench to bedside: harnessing the gut microbiota to screen out high-risk patients

In clinical practice, radiotherapy-induced toxicity depends on radiation doses, irradiated volumes of normal tissues, fractionation regimens, radiation techniques, concurrent medications and host factors [2]. Yet most animal experiments used total-body or total-abdominal irradiation at single- dose which barely resembles the clinical situations. Therefore, to validate the laboratory findings and accordingly develop predictive strategies for radiation injury risk in multiple types of cancer are necessary and translationally inspiring.

### Gynecological cancer

Two small-sample-size studies [28, 29] incorporating mixed cohorts of gynecological, colorectal and anal cancers first contributed to this field by presenting that pre-existing faecal microbiota features help identify patients predisposed to acute radiation-induced diarrhea, and that patients who prone to develop diarrhea have lower Shannon diversity index (SDI) and higher *Firmicute/Bacteroides* ratio in their baseline faecal specimens compared to those who do not. Two subsequent studies with uniform cohorts of cervical cancer patients confirmed that dysbiosis in the baseline faecal specimens from patients prone to acute RE is featured with significantly reduced α diversity (including SDI) and increased β diversity [30], and that increased SDI is an independent predictor for better near-term gastrointestinal function, when all timepoints data were analyzed together [31]. However, no association between the α diversity and occurrence of chronic RE is found in another mixed cohort of 59 patients [32], which emphasizes further microbiota exploration on the chronic RE in larger-sample-size uniform cohorts. A translational contribution deserving attention is the establishment of the first model for predicting Grade2 acute RE in a cohort of 17 gynecological cancer patients based on microbial features measured after radiotherapy [33], whose area under the receiver-operating characteristics curve (AUC) metric reaches 0.87, indicating robust predictive performance.

### *Prostatecancer*

In prostate cancer patients, Ferreira et al. [34] also observed a trend towards lower α diversity (Chao index) after radiotherapy in both acute and chronic symptomatic patients compared to their non-symptomatic counterparts despite that SDI was not analyzed. Compositionally, patients with acute and chronic RE symptoms are both rich in short chain fatty acid (SCFA)-producers, including *Clostridium IV*, *Roseburia* and *Phascolarctobacterium* whose abundances all significantly correlate positively with the elevated acute symptoms and all demonstrate more drastic post-radiotherapy decline in patients with persistent symptoms. Overabundances of *Roseburia* is even an independent predictor of occurrences of severe chronic RE. Moreover, the SCFA-producers negatively correlate with IL-15 and positively correlate with eotaxin in rectal mucosal tissues sampled from chronic RE patients, indicating an inflammation regulatory role for these microbes. Functionally, there also exists an increasing trend of butyrate metabolism and propionate metabolism associated with aggravating acute symptoms, whereas fatty acid metabolism significantly reduces with aggravating chronic symptoms.

### *Anorectalcancer*

According to a small-sample-size pilot study on rectal cancer [35], the SCFA-producing family *Lachnospiraceae* belonging to *Clostridia* class and the lactate-producing *Bifidobacterium* are overrepresented in patients with milder acute diarrhea compared to the severe counterparts. A subsequent expanded analysis showed that the microbial diversity (including SDI) negatively correlates with the severity of both myelosuppression [36] and chronic gastrointestinal toxicity significantly (data not published), and non-significantly with acute gastrointestinal toxicity [36]. Compositionally, *Akkermansia muciniphila*, *Bifidobacterium adolescentis* and *Coprococcus eutactus* are overrepresented at baseline in patients with milder myelosuppression and in patients with milder acute gastrointestinal toxicity, respectively compared to their corresponding severe groups; whereas β-glucuronidase-producing microbes including *Escherichia fergusonii* related with reactivation of the irinotecan end-product, SN38G, are overrepresented in patients with severe myelosuppression and in patients with severe acute gastrointestinal toxicity compared to the mild counterparts [36]. Established on the abundances of discriminative microbes measured prior to radiotherapy, toxicity prediction classifiers perform inspiringly with the AUC for predicting Grade2 + myelosuppression reaching 0.89, and that for predicting Grade3 + acute gastrointestinal toxicity reaching 0.86. Another study on anal squamous cell cancer [37] revealed a trend towards higher baseline microbial diversity (including SDI) in patients predisposing to Grade 2 + acute anal dermatitis compared to their Grade 0–1 counterparts, with Pielou evenness reaching significance. There is no significant association between severity of acute RE with microbial diversity. However, *Clostridia* class and *Clostridiales* order are enriched at baseline in patients with severe acute RE, which contrasts to findings in rectal cancer. Notably, this study took anal swabs from the tumor site, which is different from the faecal specimens used in other studies. Given the unique biological feature of HPV-driven anal squamous carcinoma, and the limited sequencing volume from anal swabs, as well as only 12 cases for RE analysis and 16 cases for anal dermatitis analysis, findings in this study merit further confirmation.

**Fig. 1:**
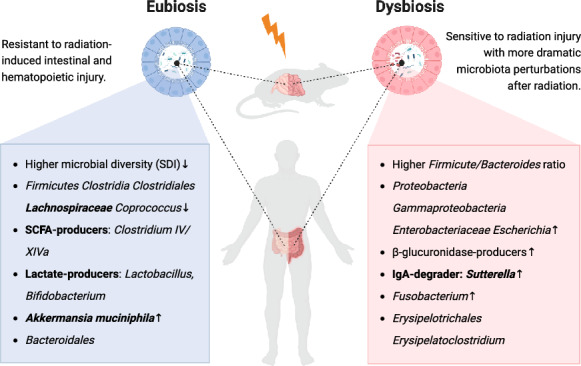
Microbiota features associated with radiation injury concordantly revealed in clinical and experimental studies. Left panel: Eubiosis associated with radio-protection phenotype in hosts, as featured by increased bacterial diversity and overrepresentation of beneficial microbes like SCFA-producers including *Lachnospiraceae* as well as *Lactobacillus* and *Akkermansia* which promote intestinal stem cell repairment by activating EGF and Wnt pathways. Dynamically, microbial diversity and *Lachnospiraceae* abundance is consistently reported to decline whereas *Akkermansia* abundance rises throughout radiotherapy. Right panel: Dysbiosis related with radiation injury vulnerability in hosts, as featured by enrichment of potentially harmful microbes including pro-diarrhea *Escherichia*, IgA-degrader *Sutterella* as well as pro-inflammatory *Fusobacterium*, whose abundances all correspondingly increase after radiotherapy. *SDI* Shannon diversity index. Created with Biorender.com

Collectively, some microbial features have been consistently identified in both animal models and clinical cohorts to be associated with amelioration of radiation injury, including lactate-producers [16, 25, 35, 36], SCFA-producers [16, 25, 29–31, 34–36] and *Akkermansia *[23, 36] (Fig. 1), as well as high α diversity [17, 20, 36] especially SDI [28, 30, 31, 33]. Nonetheless, disagreements also exist on the differential distribution of some microorganisms in different symptom groups, which will be discussed in the next section. Regarding the discrepancy on the statistical significance for α diversity, we deduce that limited sample size, as well as differences in specimen sources, study populations (varied race, gender, cancer types, etc.), methods for toxicity assessment and group stratifications may account. Summary in Table 3 may provide clues for designing confirmative studies in the future. Despite use of baseline specimens to profile the pre-existing unaffected microbiota in most clinical studies, the temporal relationship is insufficient for inferences on causality. Hence, we should interpret a particular microorganism to be beneficial or deleterious cautiously, and unravelling the molecular mechanisms is crucial.

**Table 3:** Clinical studies deciphering the predictive value of the gut microbiota in radiation-induced toxicities

References	Study population	Major techniques	Endpoints	Highlights and Comments
Manichanh, Spain, [29]	Gynecological (n = 6) or rectal cancer (n = 4) patients	Feaces for 16S rRNA DGGE + 16S rRNA sequencing	ARE (CTCAE)	The first study using 16S rRNA analysis in clinical cohorts to reveal: a) microbiota perturbations in patients with diarrhea is more drastic throughout radiotherapy; b) baseline microbiota features are distinguishable between diarrhea vs. no-diarrhea
Wang, China, [28]	Cervical (n = 8), colorectal (n = 2) and anal (n = 1) cancer patients	Feaces for 16S rRNA sequencing + blood for inflammatory markers assay	ARE (CTCAE)	The first study describing pre-treatment microbiota features predictive of acute diarrhea, including lower SDI and higher *Firmicute/Bacteroides* ratio
Wang, China, [30]	Stage II-IV cervical cancer patients (n = 18)	Feaces for 16S rRNA sequencing + coculture of colonic epithelium with faecal bacteria + blood for inflammatory marker assay	ARE (RTOG)	Baseline *Coprococcus* is enriched and microbial diversity is declined (including SDI) in patients predisposing to ARE; coculture assay demonstrated that dysbiotic microbiota from patients with severe ARE induces barrier impairment and pro-inflammatory response
Colbert, US, [31]	Stage I-IV cervical cancer patients (n = 35)	Feaces for 16S rRNA sequencing	ARE (bowel part of EPIC questionnaire, patient-reported)	High SDI independently predicts better near-term gastrointestinal function; *Clostridiales* is enriched in milder ARE and *Sutterella* in severe ARE patients
Colbert, US, [32]	Cervical, vaginal and anal cancer patients (n = 59)	Rectal swabs for 16S rRNA sequencing	CRE (RTOG and CTCAE)	Baseline *Sutterella* is underrepresented in CRE patients
Cai, China, [33]	Stage I-III cervical (n = 16) and endometrial (n = 1) cancer patients	Feaces for 16S rRNA sequencing + LC–MS	ARE (RTOG)	The first integrative multi-omics translational study constructing prediction model for ARE, using abundances of *Erysipelatoclostridium* and its downstream metabolite, ptilosteroid A
Ferreira, UK, [34]	Prostate cancer, early cohort (n = 32); late cohort (n = 87); coloscopy cohort (n = 15)	Feaces for 16S rRNA sequencing	ARE; CRE (clinician-reported: RTOG, LENT/SOM, UCLA-PCI outcomes; patient-reported: modified QoL questionnaire)	The first study reporting how gut microbiota affects CRE and emphasizing on SCFA metabolism by integration with inferred metagenomic analysis. Non-significant trend towards higher *Sutterella* exists in acute symptomatic patients
Ferreira, UK, [45]	Prostate cancer patients (n = 32)	Feaces, urine and plasma for NMR + LC–MS	CRE (patient-reported: modified QoL questionnaire)	The first study reporting microbiota-related metabolite profile associated with CRE, reinforcing the significance of SCFA in toxicity amelioration
Zhang, China, [35]	Stage I-III rectal cancer patients (n = 22)	Feaces for 16S rRNA sequencing	ARE (CTCAE)	*Clostridia*, *Bifidobacterium* and primary bile acid biosynthesis pathway are enriched in low toxicity patients
Zhang, China, [36]	Stage I-III rectal cancer patients (n = 84)	Feaces for 16S rRNA sequencing	ARE; myelosuppression (both per CTCAE)	The first study on both myelosuppression and ARE and accordingly constructing two robust prediction models. Baseline *Akkermansia*, *Bifidobacterium* and *Coprococcus* are enriched in low toxicity patients whereas β-glucuronidase-producing and pro-diarrhea *Escherichia* enriched in high toxicity patients
Colbert, US, [37]	Stage I-IV anal squamous cell cancer patients (n = 22)	Anorectal swabs at tumor site for 16S rRNA sequencing	ARE (bowel part of EPIC questionnaire); acute anal dermatitis (CTCAE)	The first pilot study focusing on both ARE and anal dermatitis

*DGGE* Denaturing gradient gel electrophoresis, *ARE* Acute radiation enteropathy, *CTCAE* Common terminology for adverse events, *SDI* Shannon diversity index, RTOG Radiation therapy oncology group scale, *EPIC* Expanded prostate cancer index composite, CRE Chronic radiation enteropathy, *LENT/SOM* Late effects of normal tissues scale, *UCLA-PCI* = Bowel problem/distress measured with the university of California, Los Angeles prostate cancer index, *QoL* = Quality of life, *SCFA* Short chain fatty acid; *NMR* Nuclear magnetic resonance, *LC–MS* Liquid chromatography-mass spectrometry

## Mechanistic investigations on microbiota-host interactions in radiation injury

Most mechanistic explorations on microbiota-host interactions in radiation injury focus on intestinal impairment, probably due to the complicated pathogenesis and limited therapeutic options in RE compared to myelosuppression. According to contemporary concept, a series of events underlie RE pathophysiology: release of abundant reactive oxygen species, enterocyte death and repairment, mucosal barrier breach, inflammation and immunosuppression, angiogenesis and vascular sclerosis as well as progressive fibrosis [2], with prostaglandin and NF-κB signaling pathways participating in all of the stages [38]. Under this frame, we summarize the mechanistic evidence for microbiota in radiation injury. Given the pathophysiological similarities between RE and IBD [2], these investigations also hold translational merits for understanding IBD pathogenesis.

### Biological functions inferred by integration with metabolomics

Unravelling the convergent biological functions behind the divergent microbial compositions may be more generalizable given the variability of microbiota profiles between individuals. In post-radiation murine plasma and urine, increased metabolism pathways include primary bile acid (BA) biosynthesis, taurine metabolism and lipid metabolism [39–41] whereas decreased metabolites include indole-3-propionic acid (IPA), 3-indoxylsulfate and indole lactate [39] which are transformed by the gut microbiota [41] from tryptophan, a type of aromatic amino acid. In irradiated murine feaces, abundances of primary BAs and taurine rise whilst abundance of secondary BAs decline with the elevated radiation dose, while aromatic amino acid metabolism and BA secretion pathways are co-activated [18]. The dramatic decline in murine faecal SCFAs [8, 42] (including acetate, propionate, butyrate and valerate) and IPA [43] after radiation which are reversed by faecal microbiota transplantation (MT) also support radio-protective attributes for these metabolites. Concordantly, in feaces from cervical cancer patients susceptible to acute RE, SCFAs demonstrate a significantly sharper decline while BAs increase after radiotherapy [44]. This finding aligns with Ferreira’s findings [34, 45] on prostate cancer that intestinal SCFA-producers and faecal butyrate level both decline more dramatically in patients with acute RE, and with Shi’s finding [35] on rectal cancer that BA biosynthesis pathway enriched in patients with milder diarrhea. Notably, butyrate alteration is not detected in plasma or urine specimens probably indicating a localized effect of this metabolite [45]. Regarding lipid metabolism, the predictive performance of the lipid-like metabolite ptilosteroid A for acute RE in gynecological cancer patients underscores its significance [33]. To summarize, post-radiation metabolomics from both rodent and human specimens consistently highlight the potential values of SCFAs and aromatic amino acid-derived indole compounds, as well as metabolism dysregulation in BAs and lipids in radiation injury.

### SCFAs and indole compounds

Both SCFAs and indole compounds are produced from gut microbiota and regarded as free-radical scavengers. SCFAs are the preferred energy source to colonocytes and serve as histone deacetylation inhibitors and G-protein-coupled-receptor (GPR) ligands. By inactivating NF-κB pathway, SCFAs upregulate anti-inflammatory cytokine IL-10, downregulate pro-inflammatory cytokine IL-6, enhance anti-microbial functions of macrophages and regulate colonic Tregs homeostasis [46]. Whether these anti-inflammatory properties contribute to radio-protection requires investigations. Fan’s group proved that butyrate attenuates post-radiation intestinal injury and inflammation, reverses dysbiosis and enhances gut integrity by activating GPR41/43 pathway [8], and that both valerate [42] and IPA [43] impart hematopoietic and gastrointestinal radio-mitigation by restoring microbial homeostasis. Mechanistically, valerate re-elevates the post-radiation decline of KRT1, a component of cytoskeleton capable of sustaining epithelial integrity against stresses whereas IPA exerts radio-protection by re-activating the down-regulated pregnane X receptor/acyl-CoA-binding protein pathway to enhance gut integrity. In parallel, selective post-radiation enrichment of *Lachnospiraceae* and *Enterococcaceae* families renders corresponding mice the “survivor phenotype” represented as attenuated hematopoietic and intestinal impairment after TBI. Butyrate and propionate derived from *Lachnospiraceae* are partly responsible by reducing DNA damage and reactive oxygen species release, and tryptophan-derived indole compounds (kynurenic acid and indole-3-carboxaldehyde) are also proved to be radio-protective albeit lacking mechanistic explanations in this study [47]. Reportedly, indole compounds as ligands to aryl hydrocarbon receptor-mediated transcriptional activity enhance proliferation of intestinal stem cells (ISCs) [48] and promote anti-oxidant and anti-inflammatory immune responses [49, 50]. One example is 3,3′-diindolylmethane which is hydrolyzed by gastric acid from indole-3-carbinol, imposes radio-protection by promoting post-radiation DNA damage repair, ISCs survival and anti-oxidative reactions as well as reversing radiation-induced gut dysbiosis [11].

### Metabolism dysregulation in BAs and lipids

BAs participate into absorption and metabolism of dietary lipids and cholesterols, and affect the detoxification of xenobiotics and the regulation of intestinal inflammation as well. Exposure of enterocytes to primary BAs aggravates inflammation in IBD whereas secondary BAs possess potent anti-inflammatory capacities [51]. The increase in primary BAs is generally attributed to post-radiation malabsorption and gut dysbiosis [18]. What deserves attention is that the beneficial *Lactobacillus* and *Ruminococcaceae* both participate into production of secondary BAs, whose post-radiation decline may therefore aggravate enteritis [18, 25]. Nonetheless, there is still a lack of direct evidence on how BA metabolism dysregulation induced by dysbiosis affects radiation injury.

Regarding the role of microbiota-regulated lipid metabolism in radiation injury, the elevated energy demand during post-radiation repairment phase may be a breakthrough although supportive evidence is lacking. Fasting-induced adipose factor (Fiaf) is an important lipid metabolism regulator secreted from epithelium which stimulates fat mobilization, triglycerides deposition, endothelial survival and vascular sprouting. According to Crawford and Gordon [4], the existence of gut microbiota suppresses the expression of Fiaf, which then increases apoptosis of endothelial cells and lymphocytes in the intestinal villi after TBI thereby aggravating enteritis and shortening post-radiation mice survival. However, no subsequent research unravels the molecular mechanism for Fiaf’s radio-protection. Another assumption is that undesirable microorganisms may radio-sensitize normal tissues by exacerbating lipid peroxidation, which is a major target for oxidative stress and radiation injury. For example, *Pseudomonas aeruginosa*, an opportunistic pathogen commonly habituating in hospitalized immunocompromised patients, induces ferroptosis, augments post-radiation gut barrier breach and ensuing lethality through producing 15-lipoxygenase. 15-lipoxygenase also promotes a transition to pro-inflammatory milieu by modulating inflammation-related lipid signaling, immunocyte recruitment and cytokine production in small intestine [52].

### Lactobacillus spp.

Experimental evidence suggests that pre-treatment with *Lactobacillus* mitigates radiation injury by improving gut dysbiosis, intestinal histopathology and by inhibiting endotoxemia occurrence and bacterial translocation [20, 53]. Radio-protective mechanisms concordantly highlight regulations of *Lactobacillus* on ISCs, which are radio-resistant and important for post-radiation gut repairment. In this process, mesenchymal cells play indispensable mediating roles by sensing microbial signals and activating downstream pathways [53–56]. According to Ciorba’s study [53, 54], gut *Lactobacillus* releases lipoteichoic acid to activate the Toll-like receptor-2 (TLR-2)/MyD88 pathway in intestinal peri-cryptal macrophages, thereby promoting the mesenchymal stem cells to migrate from villi to crypts where ISCs located, in a CXCL12-CXCR4 chemotactic manner. Lipoteichoic acid also activates TLR-2 on the migrated mesenchymal stem cells to promote the production of cyclooxygenase-2 and the ensuing synthesis of prostaglandin E_2_ (PGE_2_). Locally secreted PGE_2_ then binds E prostaglandin recepter-2 on ISCs to execute anti-apoptotic and pro-proliferative functions by activating epidermal growth factor(EGF)-Akt-Bax signaling [57]. Notably, PGE_2_ is also radio-protective of hematopoietic stem cells via activating Wnt/β-catenin pathway and of cancer cells via activating EGF-ERK2-MAPK pathway [57]. In this study, however, *Lactobacillus* renders no radio-protection on hematopoietic stem cells and even delays the growth of subcutaneously implanted colon cancer cells [54]. A possible explanation is that *Lactobacillus* functions by producing PGE_2_, which is unstable and only acts over a short distance. And hematopoietic and cancer cells are spared from exposure to PGE_2_ in gavage studies with *Lactobacillus*. In another study [55], *Lactobacillus*-derived lactate interacts with GPR81 expressed on Paneth cells and mesenchymal cells thereby increasing the secretion of Wnt3 and Wnt2b. Supply of these Wnt ligands effectively promotes β-catenin nuclear translocation in ISCs [56] and consequently improves the epithelial regeneration capacity and mice survival after exposed to radiation plus methotrexate.

However, *Bifidobacterium* also producing lipoteichoic acid and lactate did not exhibit radio-protection when administered alone according to Ciorba’s study [53], suggesting that there may exist other mechanisms as replenishment. Reportedly, *Lactobacillus* ameliorates inflammation via downstream protein p40 to promote immunoglobulin A (IgA) secretion against pathogenic infections, to reduce inflammatory cell infiltration, cytokine secretion and cytokine-induced apoptosis by activating EGF-PI3K-Akt signaling [58, 59]; and triggers anti-oxidant responses [60] by producing superoxide dismutases [61] and PGE_2_ [62]. Whether and how such anti-inflammatory and anti-oxidant properties contribute to radio-protection should be elucidated in future research.

### Akkermansia muciniphila

*Akkermansia muciniphila* residing in the mucus layer participates into the production and degradation of mucus, with SCFA as the end products. As a next-generation probiotic candidate it alleviates inflammatory disorders and metabolic syndromes and improves therapeutic responses to immune checkpoint inhibitors [63, 64]. Yet in radiation injury, the specific role of *Akkermansia* seems paradoxical at the first sight [23–25]. On the one hand, Epperly et al. reported that oral gavage of *Akkermansia* strikingly increases murine 30-day-survival rate from 0 to 100% following TBI [23]. Kim et al. [63] further demonstrated that *Akkermansia* effectively upregulates ISCs proliferation and differentiation towards secretory cells, leading to increased goblet cells and thickened mucus layer. Like lactate [55], pre-treatment with *Akkermansia* ameliorates intestinal injury in mice and organoids exposed to radiation plus methotrexate. Mechanistically, supplement of *Akkermansia* dramatically alters murine intestinal microbial composition and increases SDI, which in turn enhances the production of SCFAs. Of these, acetate and propionate effectively promote epithelial proliferation by increasing the Wnt ligands secretion from Paneth cells to activate the β-catenin-RAS-ERK signaling in ISCs. On the other hand, the expansion of *Akkermansia* is consistently observed in both radiated and colitis models [20, 23, 24], supporting this microbe in association with inflammation. Notably, Gerassy et al. [17] proved that radiation induces a proinflammatory dysbiosis featured by expansion of microbes including *Akkermansia*. Such dysbiosis is transmissible via faecal MT and enhances host secretion of IL-1β, a responsive mediator to radiation and colitis stimuli which deteriorates intestinal injury. Combining these findings, we deduce that the rise in *Akkermansia* may represent a repairment response towards the activation of inflammatory regulation, and a compensatory protection towards the shortage of SCFA-producers after radiation stimuli, rather than a deleterious factor in spite of its reported negative prognostic value in mice [25] and minipigs [21]. Given the extensive evidence in other disorders for this microbe improves prognosis by enhancing mucosal barrier and suppressing local inflammation, its role in radiation injury merits in-depth investigations.

### Cross-connections and comments for other microbes

Notably, the post-radiation decreasing trend of the order *Clostridiales* (including the family *Clostridiaceae* and the genus *Clostridium*) is concordantly reported in radiated mice [17–19, 43] and radiated patients [31, 37]. Consistent results that *Clostridia *[35] and *Clostridiales *[29–31, 47] are significantly enriched in hosts with milder post-radiation toxicities also lend strong support to this radio-sensitive microbial cluster as radio-protective, albeit with contradictory finding in one small-sample-size study investigating microbiome sampled from tumor site [37]. In literature, class *Clostridia* is perceived as beneficial by engaging into the degradation of taurine, which is a sulfur-containing amino acid involved in BA conjugation and possesses anti-oxidation and de-toxication properties [18], and *Clostridium sporogenes* is the only identified IPA-producing microbe. More importantly, bacteria in class *Clostridia* are commonly equipped with butyrate production capacity and thus involved into immunomodulation by promoting expansion of intraepithelial lymphocytes, IgA-producing cells and colonic Tregs [65]. However, *Clostridiales* is composed of mixed species including pathogens like *Clostridium perfrigens* as well. The specific radio-protective member(s) in *Clostridiales* and underlying mechanisms requires systemic investigations.

Of interest, *Coprococcus* belonging to the family *Lachnospiraceae* and the order *Clostridiales*, is significantly underrepresented at baseline in both gynecological and rectal cancer patients predisposing to acute post-radiation toxicities [30, 36], supporting this SCFA-producer being radio-protective. In prostate cancer, however, SCFA-producers were found to be overrepresented in both acute and chronic symptomatic patients throughout the treatment [32, 34]. Regarding the paradoxical finding, two interesting explanations proposed by Ferriera et al. merit validation: (1) the overrepresentation of SCFA-producers in feaces results from excessive microbe shedding secondary to the increased competition with pathogens, which is more common in patients vulnerable to radiation injury; (2) patients with pre-existing subclinical intestinal dysfunctions possess a compromised microbiota and may therefore have higher dependence on SCFA supply which is represented by the overrepresentation of SCFA-producers in their feaces, and this group may be predisposed to severe RE.

Another microorganism deserving attention is *Sutterella*, belonging to the phylum *Proteobacteria*, overrepresented in patients predisposing to acute RE following cervical or prostate radiotherapy [31, 34]. In rodent models, *Sutterella* is significantly enriched following rectum radiation and also positively correlated with colonic injury [17]. However, *Sutterella* abundance correlated inversely with occurrence of chronic RE in a mixed clinical cohort [34]. Reportedly, *Sutterella* degrades secretory IgA [66] which acts as an important local immunoregulator restricting pathogenic infections especially intracellular pathobionts like *Fusobacterium*. The *Sutterella* expansion and ensuing IgA low phenotype increases host’s susceptibility to colitis stimuli [66] and resistance to ulcerative colitis treatment [67], hence may account for the aggravation of acute RE. On the other hand, *Sutterella* possesses almost negligible capacity of promoting inflammatory cytokines production in IBD [67] which may explain its nonharmful role in the non-inflammatory pathogenesis of chronic RE although more investigations are needed to illustrate how this microbe influences radiation injury development.

**Fig. 2:**
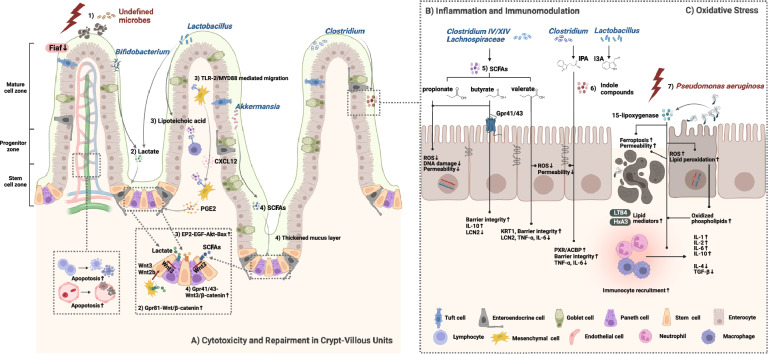
**A** Microbe-host interactions in radiation injury attenuation and augmentation. Microbes impact radiation injury development by regulating cytotoxicity and repairment in crypt-villous units: **1** Undefined microbes suppress the secretion of Fiaf from epithelium, thereby aggravating enteritis by increasing apoptosis of lymphocytes and endothelial cells in villus mesenchyme. **2** Probiotics-derived lactate interacts with Gpr81 on Paneth cells and stromal cells to stimulate secretion of Wnt3 and Wnt2b, which prompt epithelial replenishment by activating β-catenin pathway in intestinal stem cells. **3** *Lactobacillus*-derived lipoteichoic acid activates TLR-2/MYD88 on macrophages to induce secretion of chemotactic CXCL12 which binds CXCR4 to mediate the migration of mesenchymal stem cells to where intestinal stem cells locate, so as to boost enterocyte regeneration through the PGE2-EP2-EGF-Akt-Bax pathway. **4** *Akkermansia*-derived acetate and propionate bind Gpr41/43 to up-regulate the Wnt3/β-catenin-RAS-ERK pathway in intestinal stem cells, so as to promote their renewal and differentiation into secretory cells demonstrated as the thickened mucus layer. **B** SCFAs and indole compounds produced by representative microorganisms impose radio-protection through regulating inflammation and immune reseponse: **5** SCFAs promote anti-inflammation response and decrease barrier permeability by decreasing DNA damage and cell loss as well as by increasing expression of proteins related with gut integrity maintainment. **6** IPA reactivates the post-radiation declined PXR/ACBP pathway to control inflammation and improve gut integrity. **C** Microbiota-regulated oxidative stress augments radiation injury: *Psedumonas aeruginosa*-derived 15-lipoxygenase increases lipid peroxidation, induces ferroptosis and exacerbates inflammation by promoting immunocyte recruitment and elevating proinflammatory cytokines, chemokines and lipid mediators. Pro-inflammatory molecules: LCN2, TNF-α, IL-6, IL-1, IL-2, LTB4, HxA3; anti-inflammatory markers: IL-10, IL-4, TGF-β. *Fiaf* fasting-induced adipose factor, *Gpr* G-protein-coupled receptor; *TLR-2* Toll-like-receptor-2, *PGE2* prostaglandin E2, *EP2 *E prostaglandin recepter-2, *EGF* epidermal growth factor, *SCFA* short chain fatty acid, *IPA* indole-3-propionic acid, *I3A* indole-3-carboxaldehyde, *ROS* = reactive oxygen species, *LCN2* lipocalin, *KRT1* Keratin, type II cytoskeletal 1, *PXR* pregnane X receptor, *ACBP* acyl-CoA binding protein, *LTB4* leukotriene B4, *HxA3* hepoxilin A3. Created with Biorender.com

Overall, evidence supports that the gut microbiota has magnificent impacts on radiation injury via: (a) regulating cytotoxicity and repairment, and therefore sustaining epithelial and mucus barrier to defend bacterial translocation; (b) modulating host inflammation and immune functions; (c) controlling post-radiation oxidative stresses. These functional pathways are not mutually exclusive and rather occur complementally, and we briefly summarize the mechanisms in Fig. 2 and Table 4.

**Table 4:** Regulatory mechanisms of keystone microorganisms and derived metabolites in radiation injury

References	Keystone microorganism(s)	Functional constituents	Classification	Mechanism summary
Fan, China, [8]	NA	Butyrate	SCFA	Enhances barrier integrity and suppresses inflammation by activating GPR41/43 thereby ameliorating intestinal injury
Fan, China, [42]	NA	Valerate	SCFA	Enhances barrier integrity by re-elevating the post-radiation decline of cytoskeleton component KRT1, and constraints oxidative and inflammatory responses thereby ameliorating post-radiation hematopoietic and intestinal injury
Guo, US, [47]	*Lachnospiraceae*	Propionate, butyrate	SCFA	Ameliorates intestinal and hematopoietic injury by reducing release of reactive oxygen species and DNA damage
Fan, China, [43]	NA	IPA	Indole compound	Enhances barrier integrity by re-elevating the post-radiation decline of PXR/ACBP, and constraints oxidative and inflammatory responses thereby ameliorating post-radiation hematopoietic and intestinal injury
Fan, China, [11]	NA	3,3'-diindolylmethane	Indole compound	Promotes post-radiation DNA damage repairment, intestinal stem cell survival and anti-oxidative reaction thereby ameliorating post-radiation intestinal injury
Crawford and Gordon, US, [4]	NA	NA	Lipid metabolism	Existence of gut microbiota increases apoptosis and aggravates post-radiation enteritis by suppressing the expression of fasting-induced adipose factor
Dar, US, [52]	*Pseudomonas aeruginosa*	15-lipoxygenase	Lipid metabolism	Augments post-radiation intestinal barrier breach by inducing ferroptosis and promoting inflammation
Ciorba, US, [53], [54]	*Lactobacillus*	Lipoteichoic acid	Probiotics	Promotes intestinal mesenchymal stem cells to migrate to crypts to release PGE_2_ near intestinal stem cells by activating TLR2/MYD88 and CXCL12-CXCR4, so as to impart anti-apoptotic and pro-proliferative functions
Kweon, Korea, [55]	*Lactobacillus*,* Bifidobacterium*	Lactate	Probiotics	Promotes supply of Wnt ligands from Paneth cells and mesenchymal cells by activating GPR81 thereby activating β-catenin pathway and enhancing post-radiation intestinal epithelial regeneration
Kweon, Korea, [63]	*Akkermansia*	Acetate, propionate	Probiotics/SCFA	Produces SCFAs to activate Wnt/β-catenin-RAS-ERK pathway in intestinal stem cells thereby promoting proliferation and ameliorating post-radiation intestinal injury
Kaakoush, Australia, [67]	*Sutterella*	NA	NA	Degrades the immuno-protective secretory IgA thereby compromising host's anti-pathogenic capacity and possibly increasing susceptibility to injury insult like radiation, which still merits validation

*NA* Not applicable, *SCFA* Short chain fatty acid*, GPR* G-protein-coupled receptor, *KRT1* Keratin, type II cytoskeletal 1, *IPA* Indole 3-propionic acid, *PXR* Pregnane X receptor, *ACBP* Acyl-CoA-binding protein

## Microbiota manipulation strategies in radiation injury

### Faecal and oral MT

Faecal MT is established as an effective preventive option for recurrent *Clostridium difficile* infection [68] and the first product Rebyota [69] has recently been approved by FDA. However, severe complications including deaths used to be reported and data from long-term follow-up is scarce. Protocol standardization is still under cautious investigations and freshly purified materials combining multiple delivery routes are recommended for the sake of efficacy and safety [68]. In radiation injury, two research groups from China reported the feasibility of washed faecal MT in treating chronic RE, which is proven to be safer, more precise and quality-controllable than traditional crude faecal MT [70]. In Ding’s report [71], five cases aged from 45 to 81 with persistent Grade 2–4 chronic RE symptoms (per RTOG criteria) after gynecological cancer radiotherapy had at least one course of single-donor MT through the naso-jejunal transendoscopic enteral tubing. Three cases achieved the primary endpoint of ≥ one-grade reduction in toxicity at eight-week follow-up, with only one adverse event of transient nausea. However, the three cases all relapsed at longer follow-up, indicating short-lasting efficacy of this approach. In this context, strategic modifications merit consideration: (a) commencing MT earlier, which requires in advance prediction of chronic RE since this disorder is progressive; (b) repeating the courses to consolidate colonization; (c) optimizing sources of donor based on knowledge of beneficial microbiota compositions. Supportive evidence comes from Zheng’s study [72] where one 45-year-old case with continuous blood loss due to Grade 2 chronic RE (per RTOG criteria) after cervical cancer radiotherapy underwent multi-donor MT via lower-gut delivery as the first course and mid-gut delivery as the remaining three courses. At six-month follow-up, the patient’s symptoms relieved without any adverse events. This case report demonstrates that repeated transplantations of a more diverse microbiota effectively and safely mitigate hemorrhagic chronic RE, which deserves confirmation in larger cohorts with longer follow-up.

Of interest, oral microbiota is also correlated with aggravation of post-radiation oropharyngeal mucositis in nasopharyngeal cancer [73, 74], and one Chinese group successfully conducted oral MT from healthy mice to radiated mice to alleviate oral mucositis [75]. However, oral MT in colitis colorectal cancer model impaired radiotherapy efficacy and aggravated gastrointestinal toxicity, which was attributed to *Fusobacterium*, a pathogenic commensal in oral cavity [76]. Therefore, clarification of ideal consortia for mitigating toxicity is a prerequisite for application of MT in radio-protection and radio-mitigation.

### Probiotics, prebiotics and synbiotics

Probiotics containing identified live organisms may be preferrable to a mixture of uncertain components in feaces material. Lactate-producing probiotics as anti-diarrhea preventive strategy in radiotherapy patients is recommended in guidelines [77], nonetheless the low quality and heterogeneity of the analyzed clinical trials hamper its widespread application. Reports regarding influences of these probiotics on myelosuppression are scarce and debated [78, 79]. Also due to incomplete data, toxicity profile of probiotics has not been clearly summarized either. Potential risks in vulnerable population like immunocompromised cancer patients include systemic infections, deleterious metabolism, overactivated immunoregulation and gene transfer with hosts [80]. In our literature review, adverse events of probiotics use in radiotherapy patients include neutropenic infection [39], fever [78] and gastrointestinal discomforts [30] but transient and with a low incidence. Complementally, prebiotics and synbiotics also represent as viable options by fostering growth of desired microorganisms. Prebiotics containing inulin and fructo-oligosaccharide, which favor lactate-producers growth, shorten duration with watery stools but confer no significant improvements in radiation-induced symptoms in gynecological cancer patients [81]. However, synbiotics containing inulin, guar gum mixture and *Lactobacillus* reportedly attenuate acute radiation proctitis in prostate cancer patients [82]. These pilot randomized clinical trials warrant further investigations.

Up to now, some insightful implications have been accrued from probiotics practice: (a) mixture preparations outperform single-strain probiotic[83]; (b) *Lactobacillus* is preferred to *Bifidobacterium *[38, 53]; (c) prophylactic use outperforms salvage therapy [38]; (d) more prominent benefits in reducing severe diarrhea (Grade ≥ 2, per CTCAE) [84]. However, the unclear efficacy and safety require optimization of probiotics in dose, delivery and formulation. Massive mechanism-driven works also pinpoint additional radio-protective microbiota components other than *Lactobacillus* and lactate. We look forward to the development of next-generation probiotics which should be corroborated in well-designed high-quality clinical trials to change practice.

### Functional constituents, selective agonists and antagonists

The un-predictable risk with live microorganisms prompts search for pure functional constituents from microbiota as alternatives. The soluble anti-inflammatory protein p40 purified from *Lactobacillus* by Yan’s group [59] represents an example, but it is still in the laboratory stage, as are most of the keystone metabolites discussed above. Moderate-quality evidence from one small-sample-size trial suggests that sodium butyrate enema probably cannot reduce acute gastrointestinal toxicities in men undergoing prostate radiotherapy [85], which requires further investigations. Of note, lactate, SCFAs and indole compounds all possess anti-oxidant capacities. Future investigations on these metabolites will have to focus on side effects, therapeutic time window and impacts on tumor control since they constitute the major obstacles for application of anti-oxidants in radio-protection, like the case with amifostine [2].

Mechanistic exploration on microbe-host interactive pathways paves way for developing selective agonists or antagonists for radio-protection. One attractive target is TLR signaling with downstream effector PGE_2_. The TLR-4 ligand lipopolysaccharide produced by gram negative microbes [86], TLR-5 ligand derived from *Salmonella* flagellin [87, 88] and TLR-2 ligands like lipoteichoic acid from gram positive microbes [53, 54] as well as lipopeptide from *Mycoplasma* are outlooking radioprotectors [89]. Especially the engineered de-toxic derivative of *Salmonella* flagellin, Entolimod (CBLB502) is now under investigation with FDA [90]. Inhibitors of inflammatory cytokines and lipid mediators as well as microbial enzymes also promise to be tailored radio-protective strategies, including the FDA-approved IL-1β antagonist anakinra which can be administered in gut dysbiotic patients [17], and 15-lipoxygenase inhibitor baicalein which can be prescribed in *Pseudomonas aeruginosa*-infected patients [52].

Taken together, we are still at a very early stage on translating the knowledge of microbiota interacting with radiation injury into effective countermeasures. Procedure standardization and safety monitor for microbiota manipulation strategies require in-depth investigations. Both prophylactic use of probiotics and TLR agonists outperform post-radiation salvage [53, 87, 88], again highlighting the importance of prediction to tailor the radio-protective strategy according to radiation injury risk.

## Conclusions

Radiation injury is a highly complex and overlooked disorder requiring multidiscipline expertise. Symptomatic countermeasures are limitedly available at present for acute RE and myelosuppression. Physicians generally hold dismissal attitudes to chronic RE and patients tend to tolerate gastrointestinal dysfunctions lifelong. In this review, we summarized a large body of evidence for harnessing the gut microbiota to solve this challenging issue from perspectives of prediction, prevention and mitigation. Preclinical and clinical data consistently indicate some microbial signatures to be associated with both RE and myelosuppression, including lactate-producers, SCFA-producers, indole compound-producers, IgA-degraders, β-glucuronidase-producers and *Akkermansia*, etc. which not only form the basis of prediction but also provide targets for manipulation. Aside from the promising index of SDI which potentially serves as an independent toxicity predictor in multiple types of cancer, we are happy to see some inspiring pilot prediction models established using keystone microbial features. Validations of SDI and these models in carefully designed studies with larger populations are necessary. Selective MT, probiotics, purified functional metabolites and vital pathway ligands have been proposed for radio-protection and radio-mitigation, and accumulated some evidence in clinical trials. However, few of them have successfully advanced to clinical application due to nonoptimal efficacy, undesired complications and unclarified potential tumor protection. Therefore, much work remains to be done in identifying ideal radio-protective microbial consortia and optimizing microbiota manipulation strategies. To fulfill this goal, we look forward to more multi-omics data, more solid causality evidence and clarified mechanisms in the future to facilitate the customization of optimal therapeutic strategies for cancer patients undertaking radiotherapy.

## Data Availability

Not applicable.

## Abbreviations

RE: Radiation enteropathy
IBD: Inflammatory bowel disease
FDA: Food and drug administration
TBI: Total body radiation
G-CSF: Granulocyte-colony stimulating factor
SDI: Shannon diversity index
AUC: The area under the receiver-operating characteristics curve
SCFA: Short chain fatty acid
BA: Bile acid
IPA: Indole-3-propionic acid
MT: Microbiota transplantation
GPR: G-protein-coupled-receptor
ISC: Intestinal stem cells
Fiaf: Fasting-induced adipose factor
TLR: Toll-like receptor
PGE2: Prostaglandin E2
EGF: Epidermal growth factor
IgA: Immunoglobulin A
RTOG: Radiation therapy oncology group scale
CTCAE: Common terminology criteria for adverse events
